# Clinical Outcomes of Passive Sensors in Remote Monitoring: A Systematic Review

**DOI:** 10.3390/s25113285

**Published:** 2025-05-23

**Authors:** Essam Rama, Sharukh Zuberi, Mohamed Aly, Alan Askari, Fahad M. Iqbal

**Affiliations:** 1 School of Clinical Medicine, University of Cambridge, Cambridge CB2 0SP, UK; 2 Department of General Surgery, Luton & Dunstable Hospital, Luton LU4 0DZ, UK; 3 Department of Surgery, Royal Free Hospital, London NW3 2QG, UK; 4 Department of Surgery and Cancer, Imperial College London, St. Mary’s Hospital, London W2 1NY, UK; f.iqbal@imperial.ac.uk

**Keywords:** passive sensing, remote monitoring, telemedicine, wireless health, in-home monitoring, digital health

## Abstract

Remote monitoring technologies have transformed healthcare delivery by enabling the in-home management of chronic conditions, improving patient autonomy, and supporting clinical oversight. Passive sensing, a subset of remote monitoring, facilitates unobtrusive, real-time data collection without active user engagement. Leveraging devices such as smartphones, wearables, and smart home sensors, these technologies offer advantages over traditional self-reports and intermittent evaluations by capturing behavioural, physiological, and environmental metrics. This systematic review evaluates the clinical utility of passive sensing technologies used in remote monitoring, with a specific emphasis on their impact on clinical outcomes and feasibility in real-world healthcare settings. A PRISMA-guided search identified 26 studies addressing conditions such as Parkinson’s disease, dementia, cancer, cardiopulmonary disorders, and musculoskeletal issues. Findings demonstrated significant correlations between sensor-derived metrics and clinical assessments, validating their potential as digital biomarkers. These technologies demonstrated feasibility and ecological validity in capturing continuous, real-world health data and offer a unified framework for enhancing patient care through three main applications: monitoring chronic disease progression, detecting acute health deterioration, and supporting therapeutic interventions. For example, these technologies successfully identified gait speed changes in Parkinson’s disease, tracked symptom fluctuations in cancer patients, and provided real-time alerts for acute events such as heart failure decompensation. Challenges included long-term adherence, scalability, data integration, security, and ownership. Future research should prioritise validation across diverse settings, long-term impact assessment, and integration into clinical workflows to maximise their utility.

## 1. Introduction

The growing burden of chronic diseases, an ageing global population, and rising healthcare costs have intensified the need for scalable, cost-effective healthcare solutions [1]. Remote monitoring has emerged as a pivotal strategy, shifting care beyond traditional clinical settings and into patients’ homes [2]. Among these innovations, passive sensing technologies have gained traction, offering continuous, real-world health data without requiring active user engagement [3].

Passive remote integrates seamlessly into daily life through wearables, smartphones, smart home systems (e.g., Amazon Alexa, Google Nest), and ambient sensors, to collect continuous, real-world data [4,5,6]. Unlike self-reporting methods, which require user engagement and are susceptible to recall bias, passive sensing enables non-intrusive, continuous health monitoring. This is particularly beneficial for populations with cognitive impairments or conditions that hinder compliance [7,8].

These technologies—spanning Internet of Things (IoT) devices and smart home sensors—offer a rich spectrum of insights by monitoring physiological parameters, environmental conditions, and behavioural patterns [9]. Real-time or near real-time data from passive sensors holds the potential to transform healthcare by enabling early detection of clinical deterioration, facilitating timely interventions, and ultimately improving patient outcomes [3,10]. Additionally, sensor-derived data can produce digital biomarkers, providing longitudinal insights into disease progression and patient activity [7,11]. Such continuous, unobtrusive monitoring outperforms clinic-based assessments and self-reports, which are limited to intermittent snapshots and prone to subjectivity. When combined with predictive analytics and machine learning, passive sensing has the potential to shift healthcare toward a more proactive, prevention-focused model [12].

In recent years, a growing body of evidence has emerged around various remote monitoring interventions. Initial reports suggested limited clinical impact of wearable devices on individuals in the community with chronic diseases [5], but a recent analysis of digital sensor alerting systems in remote monitoring supported their use, having found a reduction in hospitalisation and in all-cause mortality [10]. Similarly, remote patient monitoring (RPM) interventions resulted in improved patient safety and functional status, and reduced cost-related outcomes such as risk of hospital readmission and length of stay [3]. Moreover, RPM was shown to reduce acute care use for patients with cardiovascular disease and COPD [13].

Despite the rapid advancement of passive sensing technologies, there is limited comprehensive evidence assessing their impact on clinical outcomes. Studies have focused on the role of smartphones-based passive sensing, with a paucity of evidence for other modalities [4,14,15]. Furthermore, there has been no analysis of the role of passive sensing. As such, the aim for this study was to synthesise current evidence on passive sensing in remote monitoring, evaluating its impact on clinical outcomes and identifying key gaps in implementation and effectiveness.

## 2. Materials and Methods

This systematic review was conducted in accordance with the Preferred Reporting Items for Systematic Review and Meta-Analysis (PRISMA) guidelines [16]. The review was registered at the International Prospective Register of Systematic Reviews (PROSPERO ID: CRD42024511755).

This review sought to answer the following questions:

Which passive sensing technologies are used in remote monitoring?

What is the impact of remote monitoring using passive sensing technologies on clinical and healthcare related outcomes?

A systematic search, with expert librarian support, was performed using the following electronic databases: Ovid in Medline, EMBASE, health management information consortium (HMIC), and PsycINFO. The search was undertaken using a list of key terms combined with the appropriate Boolean operators. Key terms included “remote”, “home”, “sens*”, “monitor*”, and “passive”. The complete search strategy is available in Appendix A. Further studies not captured by the search were identified through bibliometric cross-referencing. Grey literature was additionally searched.

All identified studies were uploaded to Covidence (Melbourne, Australia, https://app.covidence.org/sign_in, accessed on 1 August 2024), a Cochrane supported systematic review package tool [17]. Initial screening was conducted by two investigators (ER and SZ) to determine if the eligibility criteria were met. Discrepancies were resolved by discussion. Studies meeting the inclusion criteria underwent full-text screening; supplemental references were scrutinised for additional relevant articles.

Studies published detailing passive sensing technologies in the context of remote monitoring were included. The last search was performed in April 2024 with no language restrictions being placed.

Abstracts, conference articles, opinion pieces, editorials, case studies, reviews, and meta-analyses were excluded from the final review. Studies with inadequate published data relating to the outcome measures were additionally excluded.

The primary outcome measure was the impact of passive sensing technologies on clinical and healthcare-related outcomes, including early detection of health deterioration, functional monitoring, and reduction in hospitalizations. Secondary outcome measures included feasibility, usability, and adherence to passive sensing devices, as well as participant-reported challenges such as privacy concerns and technical difficulties.

All included study characteristics were extracted independently by two investigators with consensus achieved. Disagreement between the two reviewers was resolved by discussion. All full text reports of studies identified as potentially eligible after title and abstract review were obtained for further review.

Non-randomised trials were assessed using the Newcastle–Ottawa scale [18]. It comprises three variables: (i) patient selection; (ii) comparability of study groups; and (iii) assessment of outcomes. Scores range from 0 to 9: scores ≤ 3 were considered low quality, between 4 and 6 moderate quality, and ≥7 high quality. Randomised controlled trials were assessed using the Cochrane risk of bias 2 tool [19]. This assessed five possible sources of bias: (i) randomization, (ii) deviations from intended interventions, (iii) missing outcome data, (iv) measurement of outcomes, and (v) selection of the reported result. Quality assessment was performed by one reviewer (ER) and validated by a second (SZ).

## 3. Results

### 3.1. Study Characteristics

**Figure 1:**
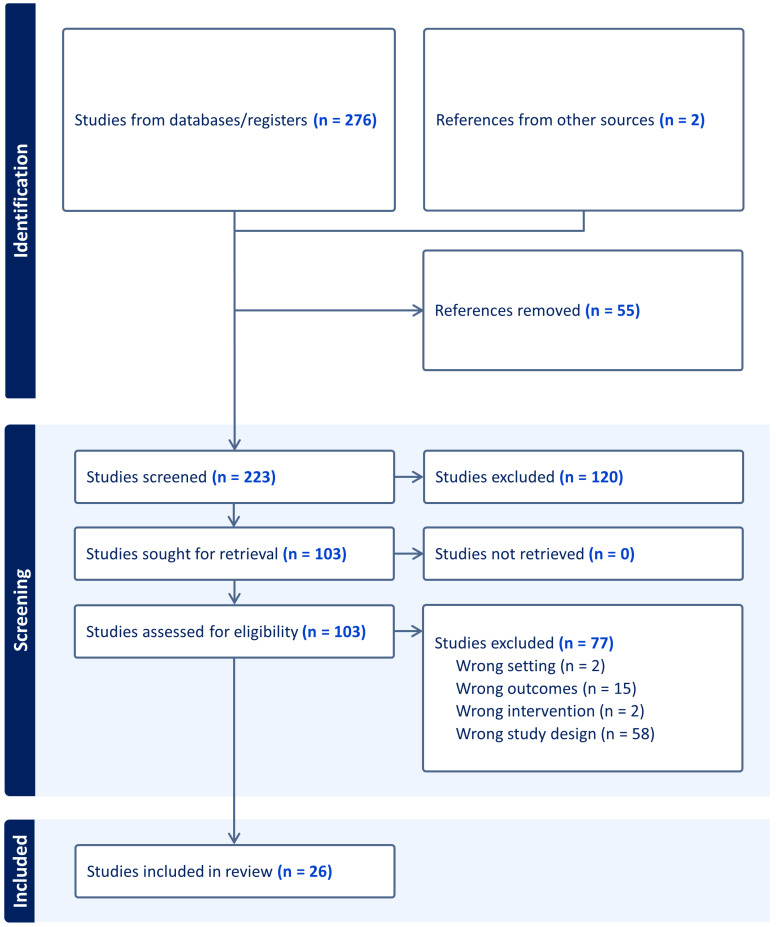
PRISMA flow diagram for included studies (Covidence).

A total of 276 results were retrieved through the literature search, of which 53 were removed due to duplications and for not meeting our eligibility criteria. Full text review was performed for 103 articles resulting in the inclusion of 26 articles in our review. The included studies were conducted in diverse global settings, including the United States (12 studies), Europe (8 studies across the United Kingdom, Germany, the Netherlands, and Switzerland), Asia (4 studies in Singapore, Japan, and China), and Australia (2 studies). The study characteristics are shown in Table 1. A PRISMA flow diagram can be seen in Figure 1.

### 3.2. Types of Passive Sensing Technology

A variety of passive sensing technologies were employed for remote monitoring (Table 2). Wrist-worn devices were the most commonly used wearables, capturing physical activity, sleep patterns, vital signs, and functional measures such as tremor severity and shoulder range of motion. Smartphones were also utilised, extending monitoring capabilities beyond activity and gait to include cognitive function, mood variability, and speech characteristics, thereby providing a more holistic assessment of patient well-being. Measures of physical activity and sleep relied principally on accelerometer and gyroscope data within smartphones and wearables.

In-home sensor systems offered additional, continuous monitoring. These included infrared cameras, infrared motion sensors, and radio-based sensors, which detected daily activity patterns, room transitions, and sleep behaviours. When integrated with door contact sensors and bed sensors, these systems provided spatiotemporally precise movement data, enhancing the characterisation of home-based activity. Infrared sensors sampled at lower frequencies (e.g., 0.5 Hz) and detected larger motions and entrances into rooms. Radio-based sensors, such as the Emerald device, transmitted low power wireless signals and analysed their reflections from nearby humans or objects using machine learning a to monitor sleep, gait speed, location, and activity. In addition to behavioural monitoring, in-home sensors also facilitated physiological assessments. Bed sensors and radio-based systems enabled the continuous tracking of respiratory rate, with deviations from baseline serving as potential indicators of clinical deterioration. Bed sensors placed underneath mattresses used highly sensitive quasi-piezoelectric films capable of measuring even slight pressure differences as produced by the beating heart (ballistocardiography).

### 3.3. Patient Populations

#### 3.3.1. Conditions Studied

The practice of remote monitoring using passive sensors occurred most commonly (six studies) in the setting of Parkinson’s disease (PD) [20,21,22,23,24,25]. Remote monitoring of older adults [8,32,33], patients with mild cognitive impairment [29,30,31], cancer patients [35,36,37], patients with musculoskeletal problems [41,42,43], and patients with cardiopulmonary conditions [38,39,40] was explored in three studies each. Dementia [27,28] was monitored in two studies. Patients with depression [34], multiple sclerosis (MS) [26], and older adults recovering from traumatic brain injury (TBI) [7] were monitored in one study each.

#### 3.3.2. Parkinson’s Disease and Multiple Sclerosis

Six studies on Parkinson’s disease [20,21,22,23,24,25] and one on multiple sclerosis [26] evaluated passive sensing for remote monitoring (Table 1). Technologies included smartwatches [25], smartphones [23,26], hybrid platforms [20,21], and in-home radio-wave sensors [22,24]. PD studies aimed to capture tremor, gait, and activity metrics, with compliance ranging from 81% for daily smartwatch wear [20] to 61% test session completion over six months [23], indicating feasibility across short and extended periods.

Algorithms processed sensor data into clinically meaningful measures, with several studies validating these against standard assessments. This involved both the conversion of raw sensor data into a clinical measure (e.g., tremor score) and the use of machine learning to categorise sensor data into activity patterns. Fay-Karmon et al. and Gatsios et al. found significant correlations between sensor-derived tremor scores and MDS-UPDRS clinician-rated measures [20,21], while both Kabelac et al. and Liu et al. demonstrated that radio-based in-home gait speed measurements aligned with MDS-UPDRS scores [22,24]. In MS, Lam et al. reported that smartphone keystroke dynamics correlated with clinical motor and cognitive tests, underscoring the potential of passive sensing for tracking disease-specific outcomes [26].

Sensor data distinguished patient and control groups, with PD participants displaying lower gait activity and slower transitions than controls [23,24], and medication-induced symptom fluctuations in PD were detectable with high temporal sensitivity [20,24]. However, predictive power for individual clinical scores remains limited, as shown by Schalkamp et al. [25], highlighting the need for further algorithm refinement.

#### 3.3.3. Dementia, Mild Cognitive Impairment, Older Adults, and Depression

Ten studies evaluated passive sensing for remote monitoring in patients with dementia [27,28], mild cognitive impairment (MCI) [29,30,31], older adults [7,8,32,33], and those with depression [34] (Table 1). Passive sensors, primarily in-home motion sensors, enabled tracking of daily activities, sleep, and vital signs, highlighting their potential in monitoring cognitive and physical decline, acute health deterioration, and functional independence.

Passive monitoring effectively captured activity patterns and cognitive function in dementia and MCI populations. Studies in MCI found subtle but informative differences in daily routines. Wu et al. observed that individuals with MCI had distinct diurnal patterns, such as increased bathroom and kitchen use compared to controls [31], while Muurling et al. noted that independent life space activity decreased among cohabiting MCI individuals [29]. Rawtaer et al. also identified trends where MCI participants had more sleep interruptions and more frequent medication lapses, suggesting changes in daily routines that passive sensing could monitor [30].

In dementia, Gaugler et al. found that activity deviations detected by sensors were communicated to caregivers, maintaining real-time awareness of patients’ well-being [27]. Similarly, Obayashi et al. showed improvements in functioning and independence among dementia patients monitored by passive sensors, with one participant even reducing reliance on night-time assistance [28]. These findings underscore the potential of passive sensors to promote independence and detect early signs of cognitive deterioration.

In older adults, passive sensing provided early detection of acute health issues and insights into health deterioration. Saner et al. reported on patients with heart failure, where episodes of decompensation were linked to reduced activity, increased heart rate, and disrupted sleep patterns [33]. Passive sensors also detected specific health events, such as pneumonia and worsening sleep disturbances [7], facilitating timely interventions. Finch et al. observed that participants with home monitoring had fewer emergency visits and hospital admissions, though cost reductions in healthcare use were not statistically significant [32].

In a study of individuals with depression, Leightley et al. noted a reduction in sleep duration during COVID-19 lockdowns, showing passive sensing’s capability to capture behavioural changes tied to mental health [34]. This study highlighted how passive sensing can monitor mood-related metrics over time, potentially aiding in the management of depressive disorders by detecting early shifts in sleep and activity patterns. Long-term continuous monitoring of such parameters may offer valuable insights and patterns of mental health conditions throughout a person’s life.

#### 3.3.4. Cancer

Three studies explored passive sensing for remote monitoring in cancer patients and survivors, using wrist-worn accelerometers, smartphones, and Fitbit devices to assess physical function and symptom burden [35,36,37] (Table 1). Two studies relied on smartphone and Fitbit data [36,37] while one utilised a wrist-worn accelerometer [35].

Low et al. examined the validity of smartphone and wearable sensors to monitor physical function in older cancer survivors, finding that greater walking duration and speed, and less fragmentation, were significantly correlated with self-reported physical function and performance measures [37]. Dadhania et al., studying high-grade glioma (HGG) patients, found similar results; daily walking time correlated positively with quality of life and physical functioning. Machine learning algorithms further identified that HGG patients exhibited half the activity levels of healthy controls, experienced longer sleep durations, and showed marked declines in moderate activity levels during radiotherapy and disease progression [35].

In a study of pancreatic cancer patients, Low et al. used machine-learning models with mobile sensor data to predict symptom burden, demonstrating higher accuracy than non-sensor-based models. Key predictors of next-day symptom burden included features related to physical activity, heart rate, and location, underscoring the value of passive sensing in anticipating symptom fluctuations [36].

#### 3.3.5. Cardiopulmonary Health

Three studies explored passive sensing for cardiopulmonary health, focusing on heart failure, hypertension, and COVID-19 recovery [38,39,40] (Table 1). Sensors included bed-based systems [38], ingestible sensors [39], and in-home radio-wave technology to capture data on respiratory rates, heart rates, body weight, and medication adherence [40].

Harrington et al. used a bed sensor to monitor heart failure patients, measuring body weight, respiratory signals, and heart rate. Validation against overnight sleep studies demonstrated close agreement with traditional respirometry and ECG, and the bed sensor effectively differentiated between central and obstructive sleep apnea. Long-term monitoring indicated that respiratory and heart rates returned to pre-surgical baselines over the course of two months, highlighting the sensor’s utility in tracking recovery [38].

Noble et al. assessed a digital health feedback system using ingestible sensors to monitor medication adherence in hypertensive patients. Over a two-week period, the system, combined with pharmacist feedback, led to a mean systolic blood pressure reduction of 7.9 mmHg, with individualised root causes of hypertension identified for each patient [39]. This approach underscores the potential of passive sensing to improve medication adherence and personalised care in hypertension management.

Zhang et al. employed a radio-wave-based in-home sensor to monitor respiratory and activity patterns in COVID-19 patients over extended periods. For one patient, a sudden respiratory rate increase coincided with hospitalisation, while stable respiratory rates in other patients indicated smooth recoveries [40]. These results demonstrate the feasibility of passive sensors in detecting early signs of deterioration during COVID-19 recovery.

#### 3.3.6. Musculoskeletal Health

Three studies assessed passive sensing in musculoskeletal health, focusing on gait quality, shoulder motion, and sit-to-stand performance [41,42,43] (Table 1). These studies utilised algorithms to convert raw sensor data into clinically relevant metrics, such as gait-quality [43], three-dimensional shoulder motion [41], and performance in the five-times sit-to-stand (5 × STS) test [42].

In patients with adhesive capsulitis, Chen et al. demonstrated that motion sensor-assisted home rehabilitation significantly improved range of motion (ROM), pain levels, and exercise adherence compared to standard home exercises, underscoring the efficacy of sensor-assisted rehabilitation [41]. Perraudin et al. used wrist-worn accelerometers to evaluate the 5 × STS test in arthritis patients over four weeks, finding that test duration was significantly linked to pain and stiffness intensity reported in patient questionnaires, suggesting that passive sensing can capture relevant functional variations in arthritis [42].

Ribeiro-Castro et al. analysed gait quality in hip and knee arthroplasty patients using smartphone-derived walking metrics, finding that early, consistent walking sessions during the first 30 days post-surgery were positively associated with improved gait speed at 90 days [43]. This highlights the potential for passive sensing to monitor recovery trajectories and optimise early post-operative activity in musculoskeletal patients.

### 3.4. Participant Feedback

Participant feedback from various studies highlighted the perceived feasibility, benefits, and challenges of passive monitoring for patients and caregivers [8,21,22,27,28,30,33,39]. Feedback revealed both positive experiences and notable concerns, providing valuable insight into the acceptability and usability of these technologies.

Some participants described passive monitoring as unobtrusive, with little impact on their daily routines [22]. However, others reported the sensors to be inconvenient or disruptive, especially when devices required direct interaction [8,30]. Preferences leaned towards non-interactive sensors, particularly for older adults and those with chronic conditions [33], indicating that usability depends heavily on device design.

Caregiver responses were mixed; some found remote monitoring helpful for managing care when living separately from patients, reducing concerns and enabling timely interventions [28,30]. In contrast, caregivers living with or providing full-time in-person care perceived less benefit from monitoring and occasionally viewed it as an additional burden [27]. Increased caregiver burden was, however, positively associated with the likelihood of monitoring use, as noted by Gatsios et al. [21], suggesting that remote monitoring may be particularly valuable in high-burden situations.

Privacy and data security emerged as concerns, with some participants expressing anxiety over being observed, which altered their behaviour [8]. While others reported minimal privacy concerns [22], ensuring data security remains important to address participant comfort with long-term use.

Some caregivers reported technical difficulties in setting up and maintaining sensor systems [27,28], underscoring the need for robust support systems to optimise usability and enhance user experience.

### 3.5. Risk of Bias Assessment

The assessment of risk of bias is tabulated in Table 3 and Table 4.

Two randomised trials were evaluated with the Cochrane risk of bias 2 tool and were deemed to be at low risk [21,27]. The quality of 24 observational studies was assessed using the Newcastle–Ottawa Scale, with scores ranging from 3 to 6, indicating a moderate to high risk of bias. Two studies achieved the highest score of 6 [23,32], whilst three studies received the lowest score of 3 [38,39,43].

Exposure ascertainment was consistently well conducted across studies. However, external validity was frequently limited due to inadequate cohort representativeness. Many studies also lacked a control group, preventing assessment of non-exposed cohorts and comparability. Additionally, for studies examining feasibility, sensor validity, or the detection of health changes, it was often challenging to confirm the absence of the outcome of interest at baseline.

Outcome assessment emerged as a methodological strength, with most studies demonstrating rigorous evaluation and adequate follow-up procedures. However, follow-up duration varied widely, and in several studies, insufficient follow-up time contributed to lower overall quality scores.

Overall, while a few studies demonstrated robust design and low risk of bias, the majority exhibited moderate quality with specific weaknesses in patient selection and confounder control.

## 4. Discussion

This review synthesises evidence exploring the clinical impact of passive sensing technologies in remote monitoring across diverse patient populations, including neurodegenerative, musculoskeletal, cognitive, and cardiopulmonary conditions. Passive sensing, through mobile devices (e.g., wearables and smartphones) and in-home systems (e.g., infrared and radio-wave sensors), demonstrated feasibility and ecological validity in capturing continuous, real-world health data. These technologies offer a unified framework for enhancing patient care through three main applications: monitoring chronic disease progression, detecting acute health deterioration, and supporting therapeutic interventions. This review demonstrates that passive sensing technologies not only correlate with established clinical assessments but also support novel, non-invasive digital biomarkers that could reshape monitoring strategies across health systems.

The scope of application spans various healthcare needs. In chronic conditions such as Parkinson’s disease and cognitive impairments, these technologies enable precise, longitudinal tracking of disease markers (e.g., tremor scores, gait speed, and daily activity patterns), facilitating timely adjustments in care. For acute health events, such as heart failure decompensation or respiratory decline during recovery from COVID-19, real-time alerts enable rapid interventions. In rehabilitation and recovery, sensor-derived feedback supports post-surgical gait improvements and adherence to physiotherapy, enhancing therapeutic outcomes.

Continuous, longitudinal monitoring enables real-time assessment of patients with chronic conditions or vulnerabilities, surpassing the limitations of infrequent clinic visits. Advances in consumer technologies, particularly smartphones, have democratised access to low-cost, high-quality sensors. Monitoring may focus on specific diseases, such as tremor assessment in PD [20], or broader functional metrics, such as sleep patterns, activity levels, or room usage in older adults [8] or those with mild cognitive impairment [30]. Strong correlations between sensor-derived metrics (e.g., tremor scores, gait speed, and sleep patterns) and established clinical assessments, underscore their potential as digital biomarkers for disease monitoring and management. Sensor-derived digital biomarkers offer unparalleled sensitivity for detecting early functional decline, facilitating disease management, and informing research [44,45]. Although many sensors have demonstrated consistent, accurate outputs under controlled conditions in preliminary testing, future research should incorporate reliability metrics in the applied setting to strengthen the validity of sensor outputs in real-world healthcare use.

Passive sensing also enables the detection of acute, clinically significant events, akin to a virtual ward model [46]. Unlike routine monitoring, this approach focuses on real-time assessment of physiological changes—such as respiratory rate (RR), heart rate (HR), and weight—coupled with digital alert systems for immediate intervention. For instance, in heart failure patients, bed sensors identified respiratory and heart rate changes predictive of decompensation [33]. Similarly, radio-wave sensors detected early respiratory deterioration in COVID-19 patients, enabling timely hospital admission [40]. These early interventions have the potential to reduce emergency visits and hospitalizations [32], though further studies are needed to evaluate scalability. A critical challenge remains in distinguishing meaningful signals from background noise. Integrating data from multiple sensor types (e.g., RR, HR, sleep patterns, physical activity) into reliable and actionable outputs is essential.

Furthermore, passive sensors can support therapeutic interventions by providing actionable feedback. For example, motion sensors improved adherence to physiotherapy exercises for shoulder rehabilitation [41] while ingestible sensors enhanced medication adherence in hypertensive patients [39]. Real-time feedback fosters a goal-oriented approach to treatment, improving patient engagement and outcomes.

This review yielded several technically significant insights. First, we identified strong, statistically validated correlations between passive sensor outputs and clinical reference standards, supporting their role as digital biomarkers. Second, multiple studies demonstrated the capacity of passive sensing platforms to deliver real-time, high-resolution data in naturalistic environments. Third, we noted the increasing integration of machine learning models, which translated raw sensor data into symptom prediction, activity clustering, and physiological state monitoring. These findings provide a foundation for future work developing standard protocols, validating predictive models, and integrating passive sensing into clinical workflows. Specifically, longitudinal studies can now build on our synthesised dataset to train and test algorithms aimed at early detection, risk stratification, and recovery monitoring.

While this review underscores the transformative potential of passive sensing technologies in remote monitoring, several limitations must be acknowledged. Firstly, the studies included in this analysis exhibit heterogeneity, encompassing diverse sensing modalities, study designs, and outcome measures. This variability complicates the synthesis of findings and limits the generalizability of the results, but for the purpose of this review, allowed for a comprehensive overview of passive sensing. For example, wearable devices predominated in some studies, focusing on gait and sleep metrics, while others relied on in-home sensors to monitor activity and physiological changes. The lack of standardisation in methodologies highlights the need for unified protocols to facilitate cross-study comparisons. Secondly, the evidence base remains constrained by the predominance of small-scale pilot studies with short follow-up durations. These exploratory investigations, while valuable for feasibility assessments, provide limited insight into the long-term clinical impact of passive sensing. Moreover, most studies were conducted in high-resource settings, raising concerns about the applicability of these findings to low-resource or underserved populations, where such technologies could have significant impact.

In addition, challenges related to usability and adherence were frequently reported, particularly with wearable devices. Despite the promise of passive sensing as a low-burden intervention, issues such as device discomfort, the need for technological proficiency, and concerns about privacy emerged as barriers to sustained use. These limitations underscore the importance of integrating patient and caregiver perspectives into the design and implementation of these technologies to improve their acceptability and usability in real-world contexts. While some studies included an analysis of patient and caregiver perspectives, future research would be enhanced by actively involving patients in the design and development of studies.

Furthermore, privacy and data security remain unresolved challenges that hinder widespread adoption. Data storage practices in longitudinal monitoring were rarely reported, highlighting an area that requires further attention and improvement. Participant discomfort with continuous monitoring, in some cases leading to altered behaviours, was a recurring theme across studies. The absence of consistent regulatory frameworks governing data protection and ethical use poses a significant barrier to the scalability of passive sensing technologies in routine clinical care. Lastly, the reliance on algorithm-driven analyses introduces inherent challenges. Although machine learning has proven effective in deriving clinically meaningful insights from sensor data, the predictive accuracy and reliability of these algorithms remain inconsistent. Many models are still in developmental stages and require further validation in diverse and real-world settings to ensure their robustness and clinical utility.

Further research, for clinicians, should focus on integrating passive sensing technologies into standard clinical workflows. This includes developing robust clinical pathways that leverage sensor-derived data to inform decision-making and enable timely interventions. Training programmes should begin to incorporate the interpretation and application of sensor outputs, ensuring that healthcare professionals can utilise these data to enhance patient care. Furthermore, clinical trials should explore the utility of passive sensors across broader patient populations and in diverse healthcare settings, particularly in resource-limited environments where access to traditional monitoring is constrained.

For academics, advancement in algorithm development and validation should be prioritised. Existing studies demonstrate the feasibility of using passive sensing to detect subtle changes in physiological and behavioural metrics, but challenges remain in achieving predictive accuracy and reliability. Future research should refine machine learning models to improve the detection of clinically significant events and symptom trajectories. One area for future development is the use of algorithms to predict symptom burden based on sensor data. For example, for cancer patients, sensors were able to predict next-day symptom burden [36]. Additionally, there is a need for longitudinal studies to evaluate the long-term impact of passive sensing on health outcomes, patient adherence, and healthcare utilisation. Collaborative efforts between academia and industry can accelerate the development of innovative sensors tailored to specific clinical needs, including multimodal devices capable of integrating physiological, environmental, and behavioural data.

Moreover, the implementation of passive sensing technologies requires clear regulatory frameworks to ensure data privacy, security, and ethical use. Addressing patient and caregiver concerns about surveillance and data handling is paramount to fostering trust and encouraging adoption. Policymakers must also consider the economic implications of these technologies, including cost-effectiveness analyses and reimbursement models that support their integration into public and private healthcare systems. Pilot programmes can evaluate the scalability of passive sensing in real-world settings and provide evidence for broader policy adoption.

In conclusion, passive sensing technologies offer a transformative approach to remote monitoring, enabling continuous, unobtrusive tracking of health metrics across diverse populations. This review underscores their potential to enhance disease management, detect early deterioration, and personalise care. However, challenges in adherence, scalability, and privacy highlight the need for further research to optimise implementation. By addressing these barriers, passive sensors could redefine clinical monitoring and support in both high- and low-resource settings.

## Figures and Tables

**Table 1:** Characteristics of included studies.

Reference	Condition or Disease	Sample Size (N)	Length of Monitoring	Sensor(s)/Feedback to Participants	Outcome Measures	Algorithms/Machine Learning	Compliance and Acceptability/Feasibility	Outcomes
Fay-Karmon et al., 2024 [20]	Parkinson’s disease	21	14 days	Smartwatch	Compliance (smartwatch usage). Smartwatch algorithm scores for tremor, dyskinesia and activity. Clinic recorded MDS-UPDRS, Daily and in-clinic motor tasks, daily symptom diary and medication intake report.	Smartwatch data as input to two algorithms: free living and motor tasks algorithm (Intel Pharma Analytics).	Compliance: 81% wearing the smartwatch for more than 12 h daily.	Validity: Significant correlation between SWA scores for tremor and both clinician assessment and MDS-UPDRS scores for tremor. Patterns: Patients categorised into four groups according to individual patterns of tremor fluctuations.
Gatsios et al., 2020 [21]	Parkinson’s disease	132	14 days	Smartphone, wristband, insoles	Compliance (protocol completion, median usage). Measures of motor and non-motor function: NMSS, UPDRS, EQ-5D-5L, PDQ-8.	An algorithm derived from a single sensor on the wrist for detecting tremor in PD patients (PD_manager).	Compliance: 85% participants completed the protocol. Median use was 11.57 days over a 14-day period.	Validity: Significant correlation between tremor score and tremor evaluation by clinicians. Significant difference between tremor score between no-tremor (UPDRS = 0) and tremor (UPDRS > 1) groups.
Kabelac et al., 2019 [22]	Parkinson’s disease	7	353 days	Radio-wave based in-home monitoring (Emerald device)	Activity: Gait speed, time in bed per day, circadian schedule (number and duration of nighttime awakenings, intraday rest-activity rhythms). Baseline MDS-UPDRS.	N/A		Validity: Significant correlation between device-derived gait speed and MDP-UDRS scores.
Lipsmeier et al., 2018 [23]	Parkinson’s disease	79 (35 controls)	6 months	Smartphone based active and passive sensing	Compliance (test session completion). Activity: algorithm determined turning speed, sit to stand transitions, proportion of gait-related activity. MDP-UPDRS during screening. Active smartphone motor testing.	Machine-learning algorithm used to classify accelerometer data into labelled activities (e.g., walking, climbing stairs, standing, and sitting).	Compliance: 61% of possible test sessions over 6 months.	Validity: All active and passive monitoring features significantly discriminated PD from controls. PD participants had less time performing gait related activities, fewer sit to stand transitions and slower 90 degree turns than controls.
Liu et al., 2022 [24]	Parkinson’s disease	50 (16 controls)	1 year (only first year included)	Radio-wave based in-home monitoring	Activity: In-home gait speed. Clinical assessment at baseline, 6 months and 12 months: MDS-UPDRS, H&Y stage, TUG, Intraday ON-OFF state from Hauser diary.	Machine learning algorithms to analyse the reflected radio signals and extracted walking movements and trajectories through the home.		Validity: Significant correlation between in-home gait speed and MDS-UPDRS and H & Y stage. Individuals with PD had about 23% lower in-home gait speed than the control cohort The decline in gait speed over one year was greater for the PD group (−0.026 m/s) compared to the control group (−0.015 m/s). Patterns: intraday gait speed oscillates according to medication administration and variation in intraday gait speed is highly correlated with patient perception of disease impact on daily function.
Schalkamp et al., 2024 [25]	Parkinson’s disease	149	485 days	Smartwatch	Activity: Weekly averages of: sleep (total time, REM time, NREM time, WASO, awakenings, sleep efficiency), physical activity (step count, walking minutes), and vital signs (pulse rate, mean RMSSD, median RMSSD, RMSSD variance). Clinic assessments: UPDRS, MoCA, LNS, HVLT, GDS, STAI, ESS, SCOPA, RBDSQ.	N/A		Validity: Digital measures capturing weekly averages of physical measures are concordant with clinical assessments of multiple non motor symptoms (cognitive, autonomic and daily functioning). Prediction: Digital weekly averages could not predict the scores of standardised non motor symptom questionnaires.
Lam et al., 2022 [26]	Multiple sclerosis	102	1 year	Smartphone	Smartphone keystroke dynamics: fine motor cluster and cognition score clusters. Standard measures: nine-hole peg test (arm function), symbol digit modalities test (information processing speed).	Keystroke features were processed to form fine motor and cognition score clusters.		Validity: The fine motor score cluster was significantly associated with the nine-hole peg test. The cognition score cluster was significantly associated with the symbol digit modalities test. Smartphone keystroke dynamics were longitudinally associated with multiple sclerosis outcomes.
Gaugler et al., 2019 [27]	Dementia	132 (64 remote monitoring, 68 usual care control)	6 months	Home activity monitors. Feedback: Significant and persistent deviations from baseline are communicated to family caregivers.	Mean caregiver self efficacy, sense of competence and distress.	Data are analysed using algorithms. Baseline activity pattern is established.		No difference in caregiver mean self efficacy, sense of competence and distress at 6 months with remote monitoring compared to control.
Obayashi et al., 2023 [28]	Dementia	5	12 weeks	Home passive infrared sensors, biometric sensors. Feedback: Alerts from the system are communicated to terminals held by care providers.	Activity: movement and sleep patterns. Weekly measures of ICF (seven measures): communication, movement, self-care, domestic activities, interpersonal activities, performing tasks (money and leisure). Weekly interRAI measurements (care measure).	N/A		Improvements in weekly measures of the International Classification of Functioning, Disability and Health (ICF) score. One participant no longer required an in-person helper at night.
Muurling et al., 2023 [29]	Mild cognitive impairment	129 homes with people living alone, 52 homes with people living as a couple	Mean of 719 days	Home passive infrared sensors, door contact sensors.	Activity: In 2-person vs. 1-person homes and MCI vs. non-MCI homes: TOOH, ILSA, kitchen, bathroom, bedroom and living room use.	Algorithms to calculate TOOH and ILSA.		Activity: People living together have a shorter TOOH, a longer ILSA, and longer room use, but only ILSA was affected by MCI status.
Rawtaer et al., 2020 [30]	Mild cognitive impairment	49 (21 controls)	2 months	Multimodal sensor system: home passive infrared motion sensors, door contact sensor, proximity beacon tags, sensor-equipped medication box, faucet usage sensor, bed sensor, wearable sensor, smart plug. Feedback: If the system detected no movement for 8 h, an alert was sent to the caregiver.	Acceptability of sensor system Activity: Daily step count, heart rate, Sleep duration, number of sleep interruptions, time away from home daily, television use. Frequency of forgetting medication, wallet and keys.	N/A	Acceptability: Sensor system was acceptable to 80% (40/49).	Activity: MCI participants were less active, had more sleep interruptions per night and forgot their medications more frequently than their cognitively healthy counterparts, although none reached significance.
Wu et al., 2022 [31]	Mild cognitive impairment	161 older adults (26 with mild cognitive impairment)	1 month	Home passive infrared sensors, door contact sensors.	Activity: trips to bathroom, bedroom, kitchen and living room. Latent trajectory models used to identify distinct diurnal patterns of room activities.	Algorithm used to extract room activities and out-of-home activities from the motion sensors.		Activity: No difference in the total number of trips to rooms at a day level between MCI and non-MCI participants. However, MCI participants were more likely to be in the high bathroom and kitchen usage groups based on diurnal patterns of activity
Finch et al., 2017 [32]	Older adults	268 (74 with passive monitoring, 2 control groups)	12 months	Home passive monitors. Feedback: Trending health alerts and real-time alerts and an emergency response pendant connecting the participant to an emergency call centre.	Activity: time in bed, toilet use, opening/closing of refrigerator, front door opening. Healthcare costs per member per month (inpatient, ED, LTC, SNF, ambulance, home care, outpatient, pharmacy, other, total). Healthcare use per member per month (acute hospitalisations, hospital days, ED visits, LTC admissions, LTC days, SNF admissions, SNF days, home care visits, outpatient visits).	Algorithms identified trends, which automatically trigger health alerts.		Healthcare costs: mean total per member per month costs were lower for the intervention group. Costs for inpatient services and ED visits were at least 10% lower in the intervention group. Home care and outpatient costs were not different. No differences were statistically significant for cost. Healthcare use: the intervention group had fewer acute hospitalisations, ED visits, long-term care admissions and days, and SNF admissions.
Lach et al., 2019 [8]	Older adults	5	3 months	Home motion sensors, bed sensor, chair sensor, door sensor, wearable sensor.	Feasibility (subjective). Activity: daily activity, sleep time and sleep efficiency, kitchen activity, bathroom activity, total activity, entry/exit, time in bed.	N/A		Validity: Sleep and activity data were similar across actigraphy and sensor measures.
Saner et al., 2020 [33]	Older adults	20	1 to 2 years	Multimodal sensor system: home passive infrared sensor, door sensor, bed sensor, wearable sensor.	Feasibility (completion). Activity: motion in the home, entry/exit, room use, sleep duration and disruption. Vital signs. Health deterioration: heart failure decompensation, palpitations, falls, pneumonia, urinary infection, pulmonary embolism. Health-related data collected from weekly visits: EQ-5D-3L questionnaire, information about accidents, information about lifestyle changes.	Sleep data extracted using algorithms.	Feasibility: 20/24 completed the study (2 died, 2 suffered health deterioration). 242,232 person hours were recorded.	Health deterioration: Episodes of heart failure decompensation were characterised by decreased activity, increased respiratory and heart rates and decreased sleep quality. Patient complaints of intermittent palpitations were confirmed by heart rate increases measured by the bed sensor.
Parkinson et al., 2023 [7]	Older adults (traumatic brain injury)	3	6 months	Home passive infrared sensor, pneumatic bed mat.	Activity: bedroom, bathroom and kitchen activity, sleep patterns and disruption. Health deterioration: pneumonia, worsening sleep disturbance.	N/A		Health deterioration/recovery: an abnormally high overnight activity correlated with an episode of pneumonia in one patient, whereas consistent sleep cycles mapped a return to work for another patient recovering from traumatic brain injury.
Leightley et al., 2021 [34]	Depression	252	9 months	Smartphone, wearable sensor (RADAR-MDD)	Sleep duration. PHQ-8 Depressive symptom questionnaire and Rosenberg self-esteem scale questionnaire.	N/A		Mean sleep duration decreased significantly between during- and post-lockdown periods. No evidence of change in depressive symptoms or self-esteem before, during, or post lockdown.
Dadhania et al., 2023 [35]	High grade glioma	42	Up to 14 weeks	Wearable sensor (wrist worn accelerometer)	Activity: mean daily activity, time spent performing functional activities (walking, light tasks, moderate activity, sedentary, sleep). PRO questionnaires: EORTC QLQ-C30, QLQ BN20, MoCA, MFI. Clinical data: MRI imaging, histopathology, radiotherapy and chemotherapy treatment plans.	Raw data classified into 5 accelerometer-predicted functional behaviours using a two-stage machine learning model.		Validity: Mean acceleration and time spent walking daily correlated positively with health quality of life and physical functioning scores and inversely with fatigue score.
Low et al., 2021 [36]	Pancreatic cancer	44	At least 2 weeks prior to surgery and 60 days after postoperative discharge.	Smartphone with AWARE app, wearable sensor.	Activity: duration of exertional and nonexertional activity and sleep. Patient-reported symptoms each morning: pain, fatigue, sleep disturbance, trouble concentrating, feeling sad, feeling anxious, shortness of breath, numbness, nausea, diarrhoea or constipation.	Models used sensor data to predict whether the next day would be a high or low symptom burden day.		Prediction: Models using LightGBM were able to correctly predict whether the next day would be a high symptom day with 73.5% accuracy, surpassing baseline models. LightGBM models predicting next-day diarrhoea (79.0% accuracy), fatigue (75.8% accuracy), and pain (79.6% accuracy) performed similarly.
Low et al., 2024 [37]	Older cancer survivors	40	4 weeks	Smartphone, wearable sensor.	Activity: daily step count, peak gait cadence, activity fragmentation, time at home, total distance travelled, geographic mobility. PRO: PROMIS-PF. Performance-based physical function: SPPB, TUG.	N/A		Validity: Wearable device features reflecting greater amount and speed and lower fragmentation of walking in daily life were significantly related to better patient-reported function and performance-based physical function. Smartphone features reflecting more geographic mobility were related to better performance-based physical function but not patient-reported function.
Harrington et al., 2021 [38]	Heart failure	Feasibility in 8 patients, long term monitoring of 1 patient.	3 months	Bed sensor (BedScales)	Overnight sleep study for validation of respiratory monitoring, heart rate monitoring and sleep study and apnoea monitoring. Long term monitoring of a heart failure patient: RR, HR, weight, presence or absence in bed.	Total body weight, respiratory waveforms, ballistocardiography quantified from force sensor data. Algorithms separate signals when the bed is shared by a partner or pet.		Validity: Close quantitative agreement of respiratory rate with measurements of belt respirometer. Heart rate monitoring showed quantitative agreement with ECG recordings. In overnight sleep studies, Bedscales could discriminate central and obstructive apneas. Health deterioration/recovery: RR and HR decreased to presurgical levels over the course of two months.
Noble et al., 2016 [39]	Hypertension	39	2 weeks	Ingestible sensor, wearable ‘patch’ sensor Feedback: Digital health feedback system: measurement of medication adherence using an ingestible sensor to inform tailored advice regarding medication or lifestyle modifications.	Serial blood pressure recordings	N/A		The mean change in SBP over the 2-week evaluation period was −7.9 ± 22.1; the mean change in DBP was −2.8 ± 12.9.
Zhang et al., 2021 [40]	COVID-19	3	327 days	Radio-wave based in-home monitoring (Emerald device)	Activity: Gait speed, sleep patterns (sleep efficiency and total time in bed), home activity patterns. Respiratory signals (RR). COVID-19 testing.	The sensor transmits wireless signals and analyses their reflections from nearby humans and inanimate objects using machine learning to infer physiological and behavioural markers.		Health deterioration/recovery: RR of patient 1 shows a smooth recovery, whereas patient 2 had a sudden increase in RR on day of hospitalisation. Asymptomatic patient 3 had a stable RR for the whole recovery period.
Chen et al., 2020 [41]	Adhesive capsulitis	15	3 months	Wearable sensors (sternum, upper arm and wrist) Feedback: Physiotherapists can view the latest shoulder ROM, exercise completion rates, can assign exercises for and directly communicate with patients through the mobile app.	Sensor measured shoulder mobility. Patient reported daily exercise completion rate. Clinic measurements of shoulder active and passive ROM by two examiners PRO: qDASH (shoulder function) and VAS (pain)	Raw data from each sensor was converted into a 3-dimensional (3D) motion of the shoulder structure	Compliance: those in the motion sensor–assisted rehabilitation group had a significantly higher patient-reported exercise completion rate.	Validity: Good to excellent reliability of the motion sensor device for measuring shoulder ROM compared to measurements of two physicians. Activity: Patients in the motion sensor-assisted rehabilitation group had significantly better ROM improvement, pain reduction and higher exercise completion rates than the home-based exercise group after three months of rehabilitation
Perraudin et al., 2018 [42]	Arthritis	45 (15 controls)	4 weeks	Wearable sensor (wrist worn accelerometer)	Adherence to STS tests 5 × STS test duration (3 times weekly) PRO questionnaires: 8 items on pain and stiffness	Algorithm to detect 5 × STS tests	Compliance: 56% of tests performed.	Validity: 5 × STS test duration was significantly associated with pain and stiffness intensity reported by patient-reported outcome questionnaires.
Ribeiro-Castro et al., 2024 [43]	Joint arthroplasty	2255	90 days	Smartphone	Activity: Gait quality metrics within 30 days of surgery. Gait speed at 90 days post surgery.	Accelerometer data from smartphone was converted into gait quality metrics		Activity: Gait speed at 90 days was significantly associated with 30-day session length in the TKA and THA cohorts Across procedure types, more evenly distributed walking bouts during the early post-operative period were positively associated with gait speed at 90 days.

Properties of included studies and their main outcomes. (5 × STS test: five-times sit-to-stand test; DBP: diastolic blood pressure, ED: emergency department; EORTC QLQ-C30: European Organisation for Research and Treatment of Cancer Quality of Life Questionnaire Core 30; EQ-5D-3/5L: EuroQol 5-Dimension 3/5-Level; ESS: Epworth Sleepiness Score; GDS: Geriatric Depression score; HC: healthy cognition; H & Y: Hoehn and Yahr stage; HVLT: Hopkins Verbal Learning Test; ICF: International Classification of Functioning; Disability and Health; ILSA: Independent Life Space Activity; InterRAI: standardised assessments; LightGBM: Light Gradient Boosting Machine; LTC: long-term care; MCI: mild cognitive impairment; MDS-UPDRS: Movement Disorder Society—Unified Parkinson’s Disease Rating Scale; MoCA: Montreal Cognitive Assessment; NMSS: Nonmotor Symptoms Scale; NREM: Non Rapid Eye Movement; PDQ-8: Parkinson’s Disease Questionnaire-8; PRO: Patient-Reported Outcomes; PROMIS-PF: Patient-Reported Outcomes Measurement Information System Physical Functioning; qDASH: Quick Disabilities of the Arm, Shoulder, and Hand questionnaire; RBDSQ: REM Sleep Behaviour Disorder Screening Questionnaire; REM: Rapid Eye Movement; RMSSD: Root Mean Squared Successive Differences (heartbeat); ROM: Range of Motion; RR: respiratory rate; SBP: systolic blood pressure; SCOPA: Scale for Outcomes in Parkinson’s Disease for Autonomic Symptoms; SNF: skilled nursing facility; SPPB: Short Physical Performance Battery; STAI: State Trait-Anxiety Inventory; SWA: Smartwatch Algorithm; TOOH: Time Out of Home; TUG: Timed Up and Go; VAS: Pain Severity Visual Analog Scale; WASO: Wake After Sleep Onset; WMS-III LNS: Letter-Number Sequencing Test).

**Table 2:** Types of passive sensors and their outputs.

Sensor Context	Type of Sensor	Sensor Data/Outcome Measure	Studies
Wearable	Wrist/Arm worn sensors (smartwatch, Fitbit, AX3 accelerometer, armband sensors)	Physical activity	[20,21,25,35]
		Step count	[25,30,36,37]
		Gait speed	[37]
		Functional behaviours	[35]
		Tremor/Dyskinesia	[20,21]
		Shoulder range of motion	[41]
		Five times sit to stand test duration	[42]
		Sleep duration	[8,21,25,34,36]
		Sleep efficiency	[8,21,25]
		Heart rate	[25,30,33,34,36]
		Respiratory rate	[33]
		Skin temperature/galvanic skin response	[33]
	Patch sensor (torso)	Step count	[39]
		Sleep duration and interruption	[39]
	Insoles	Weight bearing, balance and motion	[21]
		Gait	[21]
Ingestible	Ingestible sensor	Medication adherence	[39]
Portable	Smartphone	Activity recognition	[23,36]
		Gait features	[43]
		Speech patterns	[21,34]
		Mood	[21,34]
		Cognitive function	[21,34]
		Keystroke dynamics	[26]
		Application use	[36]
		Location/Distance travelled	[36,37]
		Light	[36]
		Screen features	[36]
In-home	Home activity monitor	Activity patterns	[8,27,32]
	Infrared camera	Activity patterns	[28]
		Sleep patterns	[28]
		Living conditions	[28]
	Infrared sensor	Room use/activity	[7,29,31,33]
		Time out of home	[29,30]
		Independent life space activity	[29]
	Radio-signals sensor	Room use/activity	[40]
		Sleep patterns	[22]
		Sleep efficiency	[40]
		Gait speed	[22,24,40]
		Respiratory rate	[40]
	Biometric sensor	Body temperature	[28]
		Heart rate	[28]
		Respiratory rate	[28]
	Door contact sensor	Time out of home	[29,30]
		Entry/exit to home	[30,33]
		Room use	[31]
		Refrigerator use	[33]
	Bed sensor	Sleep duration	[7,8,30,33]
		Sleep interruption	[7,30]
		Sleep onset delay	[33]
		Movements in bed	[7,33]
		Body weight	[38]
		Heart rate	[7,33,38]
		Respiratory rate	[7,33]
		Sleep apneas	[38]
	Chair sensor	Time on chair	[8]
	Proximity beacon	Frequency of forgetting wallet/keys	[30]
	Medication box	Frequency of forgetting medication	[30]
	Smart plug	Television use	[30]

**Table 3:** Risk of Bias assessment using Newcastle–Ottawa quality assessment scale for observational studies (★ = adequate, ☆ = not done/inadequate).

Study	Patient Selection				Comparability		Outcome			Total
	Representativeness of the Exposed Cohort	Selection of the Non-Exposed Cohort	Ascertainment of Exposure	Outcome of Interest Was Not Present at Start of the Study	Controls for Most Important Factor	Controls for Any Additional Factor	Assessment of Outcome	Follow-Up Length	Adequacy of Follow-Up Cohorts	
Chen et al., 2020 [41]	☆	☆	★	☆	☆	☆	★	★	★	4
Dadhania et al., 2023 [35]	☆	☆	★	☆	★	★	★	☆	★	5
Fay-Karmon et al., 2024 [20]	★	☆	★	☆	☆	☆	★	☆	★	4
Finch et al., 2017 [32]	★	★	★	☆	★	☆	★	★	☆	6
Harrington et al., 2021 [38]	☆	☆	★	☆	☆	☆	★	★	☆	3
Kabelac et al., 2019 [22]	☆	☆	★	☆	☆	☆	★	★	★	4
Lach et al., 2019 [8]	★	☆	★	☆	☆	☆	★	★	☆	4
Lam et al., 2022 [26]	★	☆	★	☆	☆	☆	★	★	★	5
Leightley et al., 2021 [34]	★	☆	★	☆	☆	☆	★	★	☆	4
Lipsmeier et al., 2018 [23]	☆	★	★	☆	☆	☆	★	★	★	6
Liu et al., 2022 [24]	☆	★	★	☆	☆	☆	★	★	☆	4
Low et al., 2021 [36]	★	☆	★	☆	☆	☆	★	★	★	5
Low et al., 2024 [37]	★	☆	★	☆	☆	☆	★	★	★	5
Muurling et al., 2023 [29]	★	☆	★	☆	☆	☆	★	☆	★	4
Noble et al., 2016 [39]	☆	☆	★	☆	☆	☆	★	☆	★	3
Obayashi et al., 2023 [28]	☆	☆	★	☆	☆	☆	★	★	★	4
Parkinson et al., 2023 [7]	★	☆	★	☆	☆	☆	★	☆	☆	3
Perraudin et al., 2018 [42]	☆	☆	★	☆	★	☆	★	★	☆	4
Rawtaer et al., 2020 [30]	★	★	★	☆	★	☆	★	☆	☆	5
Ribeiro-Castro et al., 2024 [43]	★	☆	☆	☆	☆	☆	★	★	☆	3
Saner et al., 2020 [33]	★	☆	★	☆	☆	☆	★	★	★	5
Schalkamp et al., 2024 [25]	★	☆	★	☆	☆	☆	★	★	☆	4
Wu et al., 2022 [31]	★	☆	★	☆	★	★	★	☆	☆	5
Zhang et al., 2021 [40]	★	☆	★	☆	☆	☆	★	★	★	5

**Table 4:** Cochrane Risk of Bias 2 assessment for randomised controlled studies (D1: Bias arising from the randomisation process; D2: Bias due to deviations from intended interventions; D3: Bias due to missing outcome data; D4 Bias in measurement of the outcome; D5 Bias in the selection of the reported result).

Study	D1	D2	D3	D4	D5	Overall
Gatsios et al., 2020 [21]	Low risk	Low risk	Low risk	Low risk	Low risk	Low risk
Gaugler et al., 2019 [27]	Low risk	Low risk	Low risk	Low risk	Low risk	Low risk

## Data Availability

No primary data were generated in this study. Data available on request.

## Appendix A

### Full Search Strategy

Full search strategy: Hillingdon Hospital Library and Knowledge Service

“Impact of passive sensing in remote monitoring—medline search”:

Ovid MEDLINE(R) ALL <1946 to 8 April 2024>

1 ((remote* or home*) adj3 (sens* or monitor*)).tw. 30,847

2 ((remote* or home*) adj3 (sens* or monitor*)).kf. 6849

3 exp Remote Sensing Technology/5244

4 1 or 2 or 3 35,793

5 passive*.tw. 143,279

6 passive*.kf. 6814

7 5 or 6 144,869

8 4 and 7 682

9 limit 8 to (English language and yr=“2014–Current” and “all adult (19 plus years)”) 64

 “Impact of passive sensing in remote monitoring—embase search”

Embase <1974 to 8 April 2024>

1 ((remote* or home*) adj3 (sens* or monitor*)).tw. 37,716

2 ((remote* or home*) adj3 (sens* or monitor*)).kf. 8115

3 exp *remote sensing/7144

4 1 or 2 or 3 42,258

5 passive*.tw. 166,882

6 passive*.kf. 12,529

7 exp *passive surveillance/71

8 5 or 6 or 7 169,585

9 4 and 8 696

10 limit 9 to (english language and yr=“2014–Current” and (adult <18 to 64 years> or aged <65+ years>)) 227

 “Impact of passive sensing in remote monitoring—emcare search”

Ovid Emcare <1995 to 2024 Week 13>

1 ((remote* or home*) adj3 (sens* or monitor*)).tw. 8712

2 ((remote* or home*) adj3 (sens* or monitor*)).kf. 1944

3 exp *remote sensing/907

4 1 or 2 or 3 9592

5 passive*.tw. 38,019

6 passive*.kf. 2569

7 exp *passive surveillance/12

8 5 or 6 or 7 38,574

9 4 and 8 159

10 limit 9 to (english language and yr=“2014–Current” and (adult <18 to 64 years> or aged <65+ years>)) 44

**Table A1:** CINAHL via Ebscohost:

S1	TI ((remote* or home*) n3 (sens* or monitor*)) OR AB ((remote* or home*) n3 (sens* or monitor*))	6699
S2	TI passive* OR AB passive*	21,467
S3	S1 AND S2	55
S6	S1 AND S2 (Limiters—Publication Date: 1 January 2014–31 December 2024 Narrow by Subject Age:—all adult Narrow by Language:—english Search modes—Boolean/Phrase)	21

**Table A2:** BNI via ProQuest.

S1	tiab((remote* or home*) near/3 (sens* or monitor*))	797
S2	MAINSUBJECT.EXACT(“Remote sensing”)	29
S3	[S1] OR [S2]	817
S4	tiab(passive*)	1974
S5	[S3] AND [S4]	6
S6	([S3] AND [S4]) AND (yr(2016) OR yr(2022))	2
S7	([S3] AND [S4]) AND (la.exact(“ENG”) AND (yr(2016) OR yr(2022)))	2

**Table A3:** Psycinfo via ProQuest:

S8	tiab((remote* or home*) near/3 (sens* or monitor*))	2309
S9	tiab(passive*)	31,580
S10	[S9] AND [S8]	64
S11	([S9] AND [S8]) AND pd(1 January 2014–9 April 2024)	48
S12	([S9] AND [S8]) AND (la.exact(“ENG”) AND pd(1 January 2014–9 April 2024))	48
S13	([S9] AND [S8]) AND (la.exact(“ENG”) AND subject.exact(“Adulthood (18 yrs & older)”) AND pd(1 January 2014–9 April 2024))	28

Cochrane Library:

ID Search Hits

#1 (((remote* or home*) near/3 (sens* or monitor*))): ti,ab,kw (Word variations have been searched) 4387

#2 MeSH descriptor: [Remote Sensing Technology] explode all trees 75

#3 #1 or #2 4390

#4 (passive*): ti,ab,kw (Word variations have been searched) 12,372

#5 (adult* or elderl* or man or men or woman or women): ti,ab,kw (Word variations have been searched) 1,006,996

#6 MeSH descriptor: [Adult] explode all trees 613,547

#7 #5 or #6 1,096,496

#8 #3 and #4 and #7 with Cochrane Library publication date Between Jan 2014 and May 2024 29

Total after deduplication in Refworks and manually screening-out remaining duplicates: 276.

Total after screening-out paediatric papers: 272.

Grey literature search via a custom search engine (created by Gavin Moore and Andrew Doyle, University Hospital Coventry and Warwickshire)
